# Development, validation, and visualization of a novel nomogram to predict stroke risk in patients

**DOI:** 10.3389/fnagi.2023.1200810

**Published:** 2023-08-07

**Authors:** Chunxiao Wu, Zhirui Xu, Qizhang Wang, Shuping Zhu, Mengzhu Li, Chunzhi Tang

**Affiliations:** ^1^Shenzhen Hospital of Integrated Traditional Chinese and Western Medicine, Shenzhen, China; ^2^Guangzhou University of Chinese Medicine, Guangzhou, Guangdong, China; ^3^Clinical Medical of Acupuncture, Moxibustion and Rehabilitation, Guangzhou University of Chinese Medicine, Guangzhou, Guangdong, China

**Keywords:** stroke, risk factors, nomogram, prediction model, NHANES

## Abstract

**Background:**

Stroke is the second leading cause of death worldwide and a major cause of long-term neurological disability, imposing an enormous financial burden on families and society. This study aimed to identify the predictors in stroke patients and construct a nomogram prediction model based on these predictors.

**Methods:**

This retrospective study included 11,435 participants aged >20 years who were selected from the NHANES 2011–2018. Randomly selected subjects (*n* = 8531; 75%) and the remaining subjects comprised the development and validation groups, respectively. The least absolute shrinkage and selection operator (LASSO) binomial and logistic regression models were used to select the optimal predictive variables. The stroke probability was calculated using a predictor-based nomogram. Nomogram performance was assessed by the area under the receiver operating characteristic curve (AUC) and the calibration curve with 1000 bootstrap resample validations. Decision curve analysis (DCA) was performed to evaluate the clinical utility of the nomogram.

**Results:**

According to the minimum criteria of non-zero coefficients of Lasso and logistic regression screening, older age, lower education level, lower family income, hypertension, depression status, diabetes, heavy smoking, heavy drinking, trouble sleeping, congestive heart failure (CHF), coronary heart disease (CHD), angina pectoris and myocardial infarction were independently associated with a higher stroke risk. A nomogram model for stroke patient risk was established based on these predictors. The AUC (C statistic) of the nomogram was 0.843 (95% CI: 0.8186–0.8430) in the development group and 0.826 (95% CI: 0.7811, 0.8716) in the validation group. The calibration curves after 1000 bootstraps displayed a good fit between the actual and predicted probabilities in both the development and validation groups. DCA showed that the model in the development and validation groups had a net benefit when the risk thresholds were 0–0.2 and 0–0.25, respectively.

**Discussion:**

This study effectively established a nomogram including demographic characteristics, vascular risk factors, emotional factors and lifestyle behaviors to predict stroke risk. This nomogram is helpful for screening high-risk stroke individuals and could assist physicians in making better treatment decisions to reduce stroke occurrence.

## Introduction

The previous stroke incidence of 12.2 million was high, and it increased to 101 million by 2019 according to the latest Global Burden of Disease Study (GBD). In addition, the GBD 2019 systematic analysis also showed that stroke was still the second leading cause of death worldwide and a major cause of long-term neurological disability, which brought an enormous financial burden to families and society (GBD 2019 Stroke Collaborators, 2021). Therefore, prevention and treatment are vital to curb the stroke pandemic. However, because of the limited effective therapies of current medical treatment and the high incidence of neuropsychiatric sequelae of stroke, stroke prevention is an effective strategy to decrease the incidence of stroke and health consequences (Boehme et al., 2017; Kleindorfer et al., 2021). Previous studies have indicated that body mass index (BMI), high blood pressure, diabetes, cardiovascular disease, and dyslipidemia are strongly correlated with the incidence of stroke (Kurth et al., 2002; Li et al., 2005; Chen et al., 2016; Du et al., 2019; Alloubani et al., 2021; Shiozawa et al., 2021). Lifestyle behaviors, including smoking, alcohol consumption, sleep quality, and depression, were also observed to be related to the occurrence of stroke (Dong et al., 2012; Christensen et al., 2018; Zhou L. et al., 2020; Luo et al., 2021). In addition, studies have also reported that basic demographic information, such as sex, age, educational level, marital status, and income level, are important factors associated with stroke (Gan et al., 2017; Jackson et al., 2018; Liu et al., 2018; Zhou W. et al., 2020). Most of the previous studies only reported one risk factor connected with stroke. The comprehensive combination of all of the above potential risk factors to predict the risk of stroke is still lacking. The occurrence of stroke is an etiologically complex disease influenced by a variety of risk factors that act and interact together and is thus not influenced by only one risk factor (Boehme et al., 2017). Therefore, constructing a predictive model that combines all potential risk factors together to detect the risk of stroke in populations is important, and it will help to detect stroke risk early and accurately and help with the adoption of appropriate preventive strategies in a timely manner.

Currently, nomograms are used as predictive tools by integrating various predictors to create graphical calculation instruments of statistical predictive models, which provide a predicted probability of a clinical event or certain endpoint outcome (Cahlon et al., 2012; Makkouk et al., 2017). Hence, constructing a nomogram is helpful to forecast the probability of stroke and provide corresponding and comprehensive preventive suggestions for individual conditions in a timely manner. However, effective and sensitive calculation models for predicting the occurrence of stroke have been scarce until now.

Therefore, in this study, we investigated the risk factors for stroke and constructed a predictive model based on the National Health and Nutrition Examination Survey (NHANES) database. First, we selected highly fitted predictor variables by least absolute shrinkage and selection operator (LASSO) and regression analysis, and then a nomogram including all potential predictors was developed and validated to forecast the probability of stroke risk. This prediction model will help to screen and identify high-risk populations for suffering stroke and provide them with optimal and timely clinical decision-making and preventive recommendations.

## Materials and methods

### Study design

This was a retrospective study, obtaining data from the NHANES (2011–2018) 8-year dataset. NHANES is an open database, with approximately 5,000 people surveyed every 2 years, represented the United States population of all ages. Prior to NHANES data collection, NHANES was approved by the Institutional Review Board (IRB) of the National Center for Health Statistics Ethics, and written informed consent was obtained from all participants.

### Study population

A total of 22,230 participants aged >20 years were selected from the NHANES 2011–2018. The inclusion criteria include the following: (1) patients with stroke (defined as participants had ever been told by a doctor or professional that they had a stroke); and (2) patients were over 20 years old. People lost clinical data or incomplete clinical information of predictor variables and outcome were excluded. Then, we cleaned the data and removed the missing data of demographic characteristics and predictor variables. Ultimately, a total of 11,435 participants were selected for our final analyses. Among them, 8531 (75%) were randomly selected using EmpowerStats software as the development group, and the remaining subjects comprised the validation group.

### Candidate predictor variables

According to the clinical experience and literature review, some variables were selected as risk predictors that might influence the occurrence of stroke, including basic demographic information (such as sex, age, educational level, marital status, income level), lifestyle behaviors (smoking, alcohol drinking, sleeping quality, and depression mood), and other highly correlated risk factors [such as BMI (underweight <18.5, normal weight > = 18.5, <25, overweight > = 25, <30, obesity > = 30), high blood pressure, diabetes, dyslipidemia and cardiovascular disease]. The basic demographic information was extracted from the self-report questionnaire and mainly included age, sex (male or female), marital status (married, divorced, separated, widowed, single, never married), educational level (less than high school, high school and more than high school), and family income level (high income > = 55,000$, 5,5000$ >mediate income > = 20,000$, lower income <20,000$).

Lifestyle behavior indexes were obtained through a questionnaire and included smoking (smoker or non-smoker), alcohol drinking status (light < = 5 alcohol drinks/day–past 12 months, 5 alcohol drinks/days <moderate < = 10 alcohol drinks/day–past 12 months, heavy >10 alcohol drinks/day-past 12 months), trouble sleeping (told doctor that they had trouble sleeping or did not tell doctor that they have trouble sleeping), and depression status [a score of > = 10 of the PHQ-9 defined as depression status, PHQ-9 < 10 defined as normal status (Kroenke et al., 2001)]. The other potential risk factors included high blood pressure (hypertension or normal), diabetes (have you ever been told by a doctor or health professional that you have diabetes, yes or no), cardiovascular disease [including ever told by a doctor had congestive heart failure (CHF), coronary heart disease (CHD), angina pectoris, myocardial infarction, yes or no] were also obtained from the questionnaire. Blood lipid data, including high-density lipoprotein [HDL, <1.00 mmol/L (low), 1.00–1.6 mmol/L (moderate), > = 1.6 mmol/L (high)], total cholesterol [TC, 0 < 5.2 mmol/L (normal), 5.2–6.2 mmol/L (borderline), > = 6.2 mmol/L (high)], were extracted from the NHANES laboratory data.

### Outcome (stroke) assessment

The endpoint outcome of our study was defined as the occurrence of stroke. The predefined stroke groups were based on the Medical Condition Questionnaire of NHANES. Participants were asked whether they had ever been told by a doctor or professional that they had a stroke. If the answer was “Yes,” then we classified these participants as the predefined stroke group. The remaining population was defined as the non-stroke group.

### Statistical analysis

Demographic data, including continuous variables and categorical variables, were extracted from the NHANES database by R 4.2.0. All continuous variables were compared between groups using the Kruskal-Wallis rank sum test and are presented as the mean ± standard deviation (mean ± SD). The chi-square test or Fisher’s exact test (if the theoretical frequency was less than 10) was performed to compare categorical variables and displayed as frequencies and proportions. Missing variable data were excluded from the analysis. A risk prediction model for stroke was constructed based on the logistic regression method. First, to avoid the collinearity of inclusion covariates and filter the optimal predicted risk factors, we screened the potential risk factors for stroke by least absolute shrinkage and selection operator (Lasso) regression. This method was helpful to avoid the collinearity of inclusion covariates and select the optimal predicted risk factors (Sauerbrei et al., 2007). The optimal features with non-zero coefficients were selected by Lasso regression and then included in a multivariable logistic regression to screen out all significant risk factors. Risk features with *P* < 0.05 were selected, and the nomogram prediction model was established. Receiver operator characteristic (ROC) curves were used to evaluate the sensitivity and specificity of the nomogram (Heagerty et al., 2000). A concordance index (C-index), calibration curves and a decision curve analysis (DCA) were used to measure the predictive performance of the nomogram. A decision curve analysis (DCA) was performed to evaluate the clinical utility of the nomogram by calculating the net benefits at different threshold probabilities (Vickers et al., 2008). To improve the accuracy and stability of the model, the 1000 bootstrap resample validation method was also conducted for internal validation. For testing validation, we performed ROC, C-index and calibration curve analyses and DCA using the same methods as mentioned above. All statistical analyses were conducted with EmpowerStats and R-4.12 software. A *p*-value of less than 0.05 indicated statistical significance.

## Results

### Baseline characteristics

A total of 11,435 participants were enrolled in this study based on the pre-specified inclusion and exclusion criteria. All included subjects had a mean age of 46.8 ± 16.9 years and included 5,959 males (52.1%) and 5,476 females (47.9%). Seventy-five percent of the eligible subjects were randomly divided into the development group (*n* = 8531), and the rest were divided into the validation group (*n* = 2904). Among the 11,435 individual subjects, 305 patients were diagnosed with stroke, 223 in the training group and 82 in the validation group. Only age, family income and hypertension status variables had significant differences between the development group and validation group (*p* < 0.05). The specific demographic information and clinical characteristics are shown in Table 1.

**TABLE 1:** Demographic data and clinical characteristics of the development and validation groups.

Group	Development group	Validation group	*P*-value
Number	8531	2904	
Age	46.6 ± 17.0	47.3 ± 16.8	0.054
**Stroke**			0.545
No	8308 (97.4%)	2822 (97.2%)	
Yes	223 (2.6%)	82 (2.8%)	
**Gender**			0.148
Male	4412 (51.7%)	1547 (53.3%)	
Female	4119 (48.3%)	1357 (46.7%)	
**Race**			0.173
Mexican American	1123 (13.2%)	355 (12.2%)	
Other Hispanic	825 (9.7%)	278 (9.6%)	
Non-Hispanic white	3568 (41.8%)	1237 (42.6%)	
Non-Hispanic black	1821 (21.3%)	610 (21.0%)	
Non-Hispanic Asian	847 (9.9%)	325 (11.2%)	
Other race-including multi-racial	347 (4.1%)	99 (3.4%)	
**Education**			0.749
Less than high school	1340 (15.7%)	473 (16.3%)	
High school	1856 (21.8%)	623 (21.5%)	
More than high school	5335 (62.5%)	1808 (62.3%)	
**Marital**			0.926
Married	4251 (49.8%)	1452 (50.0%)	
Divorced, separated, and widowed	1650 (19.3%)	574 (19.8%)	
Single	1806 (21.2%)	602 (20.7%)	
Never married	824 (9.7%)	276 (9.5%)	
**Family income**			0.017
Lower income	1777 (20.8%)	618 (21.3%)	
Mediate income	3201 (37.5%)	1006 (34.6%)	
High income	3553 (41.6%)	1280 (44.1%)	
**Alcohol drinking**			0.478
Light	7702 (90.3%)	2639 (90.9%)	
Moderate	663 (7.8%)	206 (7.1%)	
Heavy	166 (1.9%)	59 (2.0%)	
**High blood pressure**			<0.001
Yes	2775 (32.5%)	1046 (36.0%)	
No	5756 (67.5%)	1858 (64.0%)	
**Depression**			0.937
No	7819 (91.7%)	2663 (91.7%)	
Yes	712 (8.3%)	241 (8.3%)	
**Diabetes**			0.290
Yes	917 (10.7%)	332 (11.4%)	
No	7406 (86.8%)	2513 (86.5%)	
Borderline	208 (2.4%)	59 (2.0%)	
**Congestive heart failure**			0.853
Yes	199 (2.3%)	66 (2.3%)	
No	8332 (97.7%)	2838 (97.7%)	
**Coronary heart disease**			0.437
Yes	263 (3.1%)	98 (3.4%)	
No	8268 (96.9%)	2806 (96.6%)	
**Angina/Angina pectoris**			0.550
Yes	177 (2.1%)	55 (1.9%)	
No	8354 (97.9%)	2849 (98.1%)	
**Trouble sleeping**			0.390
Yes	2318 (27.2%)	813 (28.0%)	
No	6213 (72.8%)	2091 (72.0%)	
**Smoking**			0.578
Yes	4005 (46.9%)	1346 (46.3%)	
No	4526 (53.1%)	1558 (53.7%)	
**BMI**			0.712
Underweight	112 (1.3%)	45 (1.5%)	
Normal weight	1305 (15.3%)	457 (15.7%)	
Overweight	2498 (29.3%)	835 (28.8%)	
Obesity	4616 (54.1%)	1567 (54.0%)	
**HDL (mmol/L)**			0.121
<1.00 (low)	1329 (15.6%)	408 (14.0%)	
1.00–1.6 (moderate)	4893 (57.4%)	1681 (57.9%)	
> = 1.6 (high)	2309 (27.1%)	815 (28.1%)	
**TC (mmol/L)**			0.495
5.2 mmol/L (normal)	5322 (62.4%)	1788 (61.6%)	
1 5.2–6.2 (borderline)	2231 (26.2%)	760 (26.2%)	
2 > = 6.2 (high)	978 (11.5%)	356 (12.3%)	

### Clinical predictor selection

**FIGURE 1:**
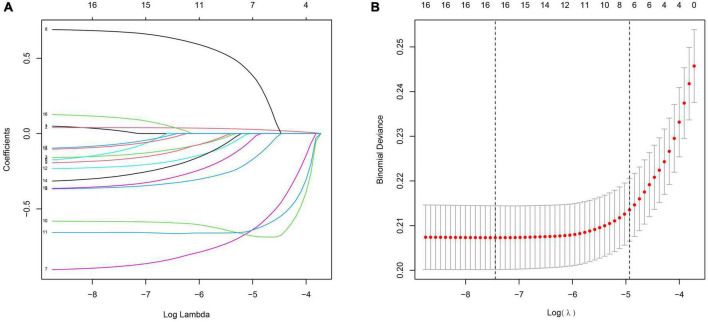
Clinical feature selection using the Lasso logistic regression model. **(A)** The partial likelihood deviance (binomial deviance) curve was plotted vs. log (lambda). Dotted vertical lines were presented at the optimal predictors using the minimum criteria (min.criteria) and the 1 SE of the minimum criteria (1-SE criteria). **(B)** Lasso coefficients of a total 17 clinical features.

According to clinical experience, expert opinion and previous literature, we included 17 potential predictors in the Lasso logistics analysis to avoid overfitting of the variables and enhance the accuracy of the prediction model. Finally, a total of 16 variables were left based on the minimum criteria of non-zero coefficients (Figure 1). Twelve risk factors with *p*-values less than 0.05 were further selected by logistic regression [including age, education, family income, hypertension, depression status, diabetes, alcohol, smoking, trouble sleeping, coronary heart disease (CHD), angina pectoris and myocardial infarction; Table 2].

**TABLE 2:** Screening out risk factors for stroke by logistic regression.

Variables	β (95% CI)/OR (95% CI)	*P*-value
Age	1.06 (1.05, 1.07)	<0.0001
**Gender**		
Male	Ref	
Female	1.01 (0.81, 1.27)	0.9129
**Education**		
Less than high school	Ref	
High school	0.88 (0.64, 1.20)	0.4172
More than high school	0.49 (0.37, 0.65)	<0.0001
**Family income**		
Lower income	Ref	
Mediate income	0.66 (0.51, 0.85)	0.0016
High income	0.30 (0.22, 0.41)	<0.0001
**Alcohol**		
Light	Ref	
Moderate	0.53 (0.30, 0.93)	0.0272
Heavy	0.80 (0.33, 1.95)	0.6173
**High blood pressure**		
Yes	Ref	
No	0.17 (0.13, 0.22)	<0.0001
**Depression**		
No	1	
Yes	3.00 (2.25, 3.99)	<0.0001
**Diabetes**		
Yes	Ref	
No	0.28 (0.21, 0.36)	<0.0001
Borderline	0.85 (0.49, 1.48)	0.5667
**Congestive heart failure**		
Yes	Ref	
No	0.11 (0.08, 0.16)	<0.0001
**Coronary heart disease**		
Yes	Ref	
No	0.13 (0.09, 0.18)	<0.0001
**Angina/angina pectoris**		
Yes	Ref	
No	0.16 (0.11, 0.24)	<0.0001
**Trouble sleeping**		
Yes	Ref	
No	0.41 (0.33, 0.52)	<0.0001
**Smoking**		
Yes	Ref	
No	0.46 (0.36, 0.58)	<0.0001
**BMI**		
Underweight	Ref	
Normal weight	0.85 (0.33, 2.17)	0.7361
Overweight	0.62 (0.25, 1.57)	0.3161
Obesity	0.94 (0.38, 2.31)	0.8888
**HDL (mmol/L)**		
<1.00 (low)	Ref	
1.00–1.6 (moderate)	0.75 (0.54, 1.03)	0.0717
> = 1.6 (high)	1.06 (0.76, 1.49)	0.7288
**TC (mmol/L)**		
<5.2 (normal)	Ref	
5.2–6.2 (borderline)	0.84 (0.64, 1.11)	0.2209
> = 6.2 (high)	0.81 (0.55, 1.19)	0.2907

### Development and assessment of the predictive nomogram

#### Model development

**FIGURE 2:**
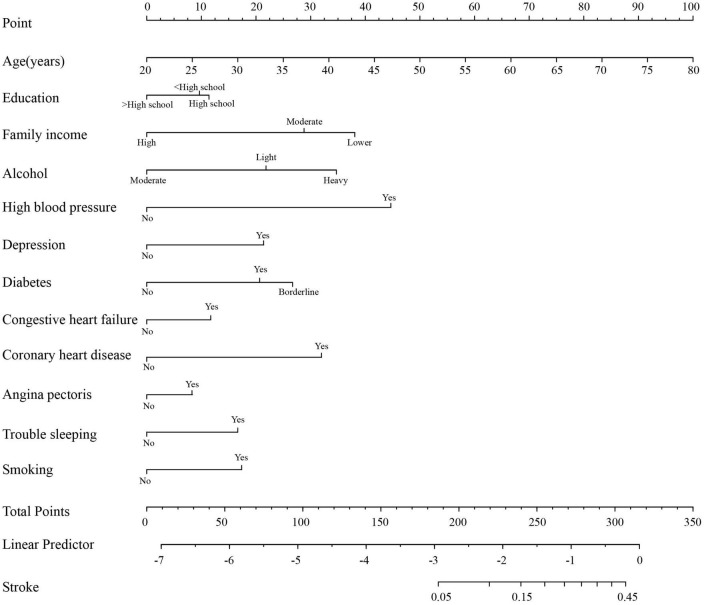
Nomogram to predict the probability of stroke in patients.

**FIGURE 3:**
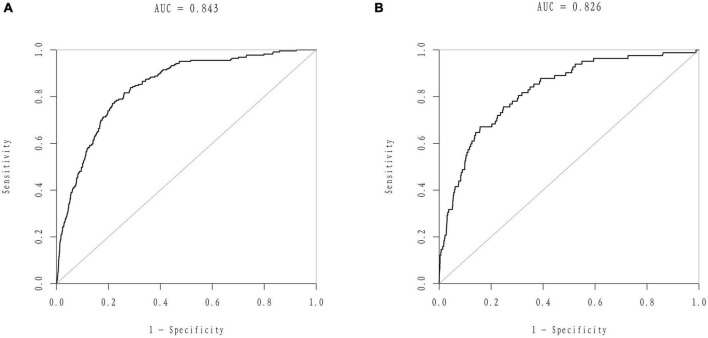
Receiver operator characteristic (ROC) curves of the nomogram. **(A)** ROC curves of the nomogram in the training group. **(B)** ROC curve of the nomogram in the validation group.

**FIGURE 4:**
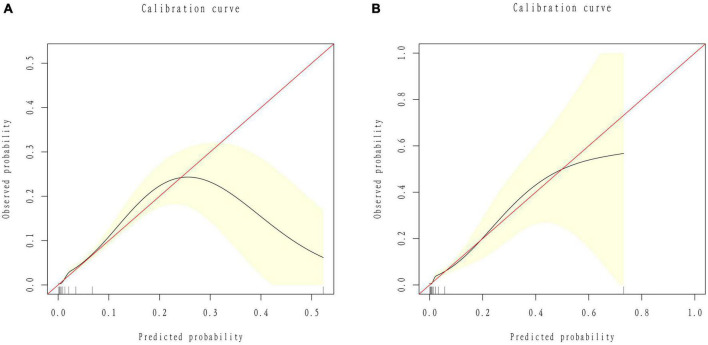
Calibration curves of the nomogram. **(A)** ROC curves of the nomogram in the training group. **(B)** ROC curve of the nomogram in the validation group.

**FIGURE 5:**
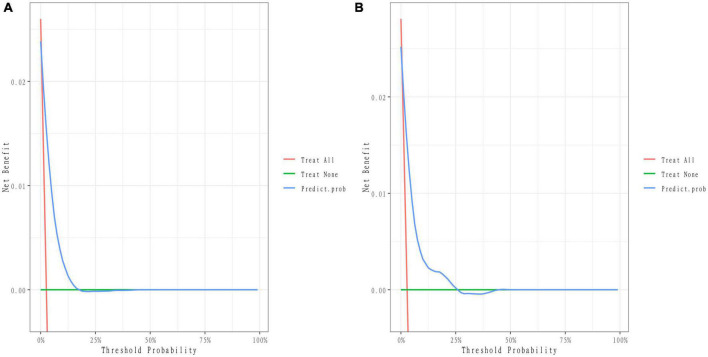
Decision curve of the nomogram. **(A)** Decision curves of the nomogram in the training group. **(B)** Decision curves of the nomogram in the validation group.

We developed a predictive full nomogram containing age, educational level, family income, hypertension, depression status, diabetes, cardiovascular disease [including congestive heart failure (CHF), coronary heart disease (CHD), angina pectoris, myocardial infarction], trouble sleeping, smoking, and drinking based on the minimum criteria of non-zero coefficients of Lasso regression and significant logistic regression screening. Each predictor was calculated as a specific score on a rating scale, the total points of each variable were summed, and a vertical line was drawn downward at the total points to correspond to the probability of stroke. A higher score indicated a higher probability of stroke (shown in Figure 2). The area under the ROC curve [AUC (C statistic)] of this full nomogram was 0.843 (95% CI: 0.8186–0.8430; Figure 3A). The calibration curves after 1000 bootstraps displayed a good fit between the actual and predicted probabilities in the nomogram, which indicated that the predictive models were stable and accurate (Figure 4A). The curves showed that the model had a net benefit when the risk threshold was between 0 and 0.2 (Figure 5A).

#### Model validation

The 25% random internal validation indicated that these nomogram models had good predictive performance and good stability. The AUC (C statistic) of the model in the validation cohort was 0.826 (95% CI: 0.7811, 0.8716) (Figure 3B). The nomogram calibration curve of the validation cohort also demonstrated that the model had good calibration (Figure 4B). The decision curves showed that the model had a net benefit when the risk threshold was between 0 and 0.25 (Figure 5B).

## Discussion

Given the high occurrence rate of stroke and heavy burden of post-stroke sequelae, an optimal prediction model for stroke prevention is clinically meaningful. Therefore, in this study, we screened the potential risk factors by Lasso and logistic regression and constructed and validated a novel nomogram-based score including these competing risk factors to predict the risk of suffering stroke.

Our results revealed that patients with older age, lower education level, lower family income, hypertension, depression status, diabetes, heavy smoking, heavy drinking, trouble sleeping, myocardial infarction congestive heart failure (CHF), coronary heart disease (CHD), angina pectoris and myocardial infarction had a higher risk of developing stroke. Similar to the reports of previous studies, older age, hypertension and diabetes were significantly and highly associated with stroke (Hu et al., 2005; Alloubani et al., 2018; Yousufuddin and Young, 2019).

Aging increases susceptibility to cerebrovascular diseases and increases the complications of stroke with the decline of physical function (Boehme et al., 2017; Yousufuddin and Young, 2019). Hypertension and diabetes mainly affect vascular blood flow and vascular function, which are positively associated with stroke mortality and morbidity (Sowers et al., 2001; Chang et al., 2022). Heart disease, including coronary heart disease (CHD) and myocardial infarction, was a confirmed risk factor and was considered to be 3–4 times more prevalent than the absence of heart disease (Arboix, 2015; Virani et al., 2021). These results further validated that aging, hypertension, diabetes and heart disease were associated with a higher risk for developing stroke. This study also indicated that individuals with lower family income had a higher risk for stroke than those with higher income. Related studies were in line with our study and suggested that lower income was strongly related to stroke occurrence and its risk factors, including hypertension and smoking (Virani et al., 2021). Heavy smoking is a dangerous risk factor for stroke, as previously reported. Most of the studies suggested that an increment of five cigarettes would increase the risk of stroke by 12% according to the dose-response analysis or that heavy cigarette consumption (5 or more cigarettes per day) would increase the risk of developing stroke (Pan et al., 2019; Luo et al., 2021). Regarding trouble sleeping, most studies have demonstrated that long-term sleep disturbance causes endocrine abnormalities that directly or indirectly influence blood pressure, glucose, and lipids, resulting in cerebrovascular pathology of stroke (Grandner et al., 2016; Phua et al., 2017). Emotional behavior also influences the occurrence of stroke, and our study indicated that depression had a positive association with a high risk of developing stroke, which was consistent with previous study analyses (Li et al., 2021). Depression might increase stroke risk by affecting neuroendocrine and inflammatory responses and unhealthy behaviors (heavy smoking and drinking, irregulated diet) (Strine et al., 2008; Pan et al., 2011; Shen et al., 2022).

We used Lasso regression to build an optimized nomogram-based model. The AUC results in the training and validation groups were 0.843 and 0.825, respectively, indicating that the nomogram predictive model had good accuracy and stability. This nomogram model was convenient to use because the number of predictors was smaller and the calculation methods were easy to master. This nomogram, including demographic characteristics, vascular risk factors, emotional factors and lifestyle behaviors, is comprehensive and sensitive for identifying high-risk individuals who have not yet developed stroke. Previous studies indicated that stroke occurrence was not only one risk factor but also influenced by multiple factors that interact with each other (Boehme et al., 2017). Nomograms are helpful for predicting the probability of suffering from disease (Park, 2018). Previous studies have shown that nomograms are widely used in the prediction of stroke, including establishing prediction models to identify blood biomarkers or medical imaging data associated with stroke and stroke implications (Liu J. et al., 2022; Liu L. et al., 2022; Yang et al., 2022). All these results indicated that the nomogram is stable and accurate in predicting the probability of suffering stroke, which was in line with our study.

Our nomogram model can obtain the total score of risk factors and the risk probability of developing stroke, which can help physicians make more helpful suggestions for patients. For instance, for an individual aged 60 years who had a lower education and family income, hypertension, depression status, diabetes, heavy smoking, heavy drinking, trouble sleeping, coronary heart disease (CHD), congestive heart failure (CHF) and myocardial infarction (angina pectoris), the total score of risk factors provided by the nomogram model is 355, which corresponds to a 75% probability of stroke. Therefore, the physician could judge that this patient has a high risk of developing stroke and suggest that this patient actively control risk factors by lifestyle and behavior change and take corresponding treatment measures to reduce stroke risk.

To our knowledge, previous studies also established predictive model to predict the stroke risk. All previous prediction models including Framingham Stroke Risk Score, CHADS2 and CHA2DS2-VASc Score, were stable and accurate in predicting the probability of suffering stroke, which were consistent with our results. However, the Framingham Stroke Risk Profile (FSRP) that only includes predictors such as age, systolic blood pressure, antihypertensive treatment, diabetes history, smoking, and cardiovascular disease history to predict the stroke risk in future 10 years (Wolf et al., 1991). Therefore, the model may have some limitations in that it lacks some important stroke predictors. Other prediction models including CHADS2 and CHA2DS2-VASc Score mainly assessed the incidence of stroke in patients with non-valvular atrial fibrillation, which could better guide stroke risk stratification and anticoagulant drug application in patients with atrial fibrillation (Gage et al., 2001; Lip et al., 2010). These models could only apply in atrial fibrillation people which might narrow the scope of application of the population compared with our prediction model. Therefore, in this model, we comprehensively included potential risk factors and established a predictive model by a visual graphical calculation instrument to forecast the probability of stroke.

However, there are still several limitations of our prediction model. First, our nomogram model was based on the NHANES database, indicating that the data were retrospective, and some variables were based on self-report, which increased the selection bias and reduced the accuracy of our model. We established the prediction model from random and different time points. Second, because of the limited data of NHANES, some potential pathogenic gene statuses, etiological subtypes, imaging results and other important risk factors were not investigated to further establish the comprehensive prediction model. Third, this model was mainly established based on the American population, and multicenter clinical validation is needed to further evaluate the external applicability of this nomogram model.

## Conclusion

This study established a nomogram that included demographic characteristics, vascular risk factors, emotional factors and lifestyle behaviors to predict the risk of developing stroke. The validations also showed the accurate and stable predictive performance of this nomogram. This nomogram is helpful to screen high-risk stroke individuals and could assist physicians in making better treatment decisions to reduce stroke occurrence.

## Data availability statement

The datasets presented in this study can be found in online repositories. The names of the repository/repositories and accession number(s) can be found below: https://www.cdc.gov/nchs/nhanes/.

## Ethics statement

The studies involving human participants were reviewed and approved by the Institutional Review Board (IRB) of the National Center for Health Statistics Ethics. The patients/participants provided their written informed consent to participate in this study.

## Author contributions

CW and CT designed the study. ZX, SZ, and ML acquired the data. CW and QW analysis and interpreted the data. CW drafted the manuscript. QW and CT revised the manuscript for important intellectual content. All authors read and approved the final manuscript.
